# Supplementary material for: Resolving the full spectrum of human genome variation using Linked-Reads
Source: Genome Res. 2019 Apr;29(4):635–45. doi: 10.1101/gr.234443.118 (PMC6442396; doi:10.1101/gr.234443.118)
Supplement: Supplemental Material [file supp_gr.234443.118_Supplemental_longranger-2.2.zip › longranger-2.2.tar.bz2!/lr2.2-public/lib/stan/cmdstan/stan/doc/api/html/rev_2operators_2operator__equal_8hpp_source.html]

Stan: src/stan/agrad/rev/operators/operator\_equal.hpp Source File


|  |  |
| --- | --- |
|  | Stan  2.5.0  probability, sampling & optimization |


- Main Page
- Related Pages
- Namespaces
- Classes
- Files

- File List
- File Members

All Classes Namespaces Files Functions Variables Typedefs Enumerations Enumerator Friends Macros Pages

- src
- stan
- agrad
- rev
- operators

operator\_equal.hpp

Go to the documentation of this file.

1 #ifndef STAN\_\_AGRAD\_\_REV\_\_OPERATORS\_\_OPERATOR\_EQUAL\_HPP

2 #define STAN\_\_AGRAD\_\_REV\_\_OPERATORS\_\_OPERATOR\_EQUAL\_HPP

3

4 #include <stan/agrad/rev/var.hpp>

5

6 namespace stan {

7  namespace agrad {

8

26  inline bool operator==(const var& a, const var& b) {

27  return a.val() == b.val();

28  }

29

39  inline bool operator==(const var& a, const double b) {

40  return a.val() == b;

41  }

42

51  inline bool operator==(const double a, const var& b) {

52  return a == b.val();

53  }

54

55  }

56 }

57 #endif

stan::agrad::var::val

double val() const

Return the value of this variable.

**Definition:** var.hpp:209

stan::agrad::operator==

bool operator==(const fvar< T > &x, const fvar< T > &y)

**Definition:** operator\_equal.hpp:14

var.hpp

stan::agrad::var

Independent (input) and dependent (output) variables for gradients.

**Definition:** var.hpp:27


---

[ Stan Home Page ]

*© 2011–2014,
Stan Development Team.*
